# Cognitive Aging Among First-Generation College Graduates and Those with Different Educational Backgrounds

**DOI:** 10.1093/geroni/igaf122.2556

**Published:** 2025-12-31

**Authors:** Masahiro Toyama, Lexi Giroski

**Affiliations:** Marshall University, Huntington, West Virginia, United States; Marshall University, Huntington, West Virginia, United States

## Abstract

Research is lacking on first-generation college students’ postgraduate outcomes, particularly aging-related cognitive outcomes. While previous research suggested the positive implications of one’s own and/or parents’ education for cognitive aging, the present study aims to uniquely contribute to the literature by contrasting aging first-generation college graduates (FGCGs) and others with different educational backgrounds. We specifically examined whether college graduation predicted better cognitive outcomes and whether the results of FGCGs were comparable to continuing-generation college graduates (CGCGs; having parent(s) with college education). We analyzed two-wave data from the Cognitive Projects of the Midlife in the United States (MIDUS) (N = 4,206 at Time 1: mean age = 56.0, SD = 12.3; 54% female) while including education variables from the MIDUS main surveys. Our multiple regression analyses indicated that participants’ college graduation, but not parents’ college education, predicted verbal memory at Time 1 and its residualized change over a decade (i.e., Time 2 verbal memory controlling for its Time 1/baseline levels). In contrast, both participants’ college graduation and parents’ college education predicted Time 1 executive function, though neither predicted its change. There was an interaction effect, indicating that Time 1 executive function did not differ between FGCGs and CGCGs, while it differed by their parents’ education among non-college graduates. In other words, the advantage of having college-educated parents appeared limited to those who did not graduate from college. By showing comparable results between FGCGs and CGCGs, this study highlights the importance of completing college education, particularly for those whose parents lacked college education.

